# Comprehensive genomic survey, structural classification and expression analysis of C_2_H_2_-type zinc finger factor in wheat (*Triticum aestivum* L.)

**DOI:** 10.1186/s12870-021-03016-3

**Published:** 2021-08-18

**Authors:** Yongliang Li, Aolong Sun, Qun Wu, Xiaoxiao Zou, Fenglin Chen, Ruqiong Cai, Hai Xie, Meng Zhang, Xinhong Guo

**Affiliations:** College of Biology, Hunan University, Changsha, 410082 China

**Keywords:** *Triticum aestivum*, C_2_H_2_-ZFP, Molecular structural analysis, Subet-specific motifs, Expression patterns

## Abstract

**Background:**

The C_2_H_2_-type zinc finger proteins (C_2_H_2_-ZFPs) are one of major classes of transcription factors that play important roles in plant growth, development and stress responses. Limit information about the *C*_*2*_*H*_*2*_*-ZF* genes hinders the molecular breeding in bread wheat (*Triticum aestivum*).

**Results:**

In this study, 457 C_2_H_2_-ZFP proteins (including 253 splice variants), which contain four types of conserved domain (named Q, M, Z, and D), could be further classified into ten subsets. They were identified to be distributed in 21 chromosomes in *T. aestivum*. Subset-specific motifs, like NPL-, SFP1-, DL- (EAR-like-motif), R-, PL-, L- and EK-, might make C_2_H_2_-ZFP diverse multifunction. Interestingly, NPL- and SFP1-box were firstly found to be located in C_2_H_2_-ZFP proteins. Synteny analyses showed that only 4 pairs of C_2_H_2_ family genes in *T. aestivum*, 65 genes in *B. distachyon*, 66 genes in *A. tauschii*, 68 genes in rice, 9 genes in *Arabidopsis*, were syntenic relationships respectively. It indicated that *TaZFPs* were closely related to genes in *Poaceae*. From the published transcriptome data, totally 198 of 204 *TaC*_*2*_*H*_*2*_*-ZF* genes have expression data. Among them, 25 *TaC*_*2*_*H*_*2*_*-ZF* genes were certificated to be significantly differentially expressed in 5 different organs and 15 different development stages by quantitative RT-PCR. The 18 *TaC*_*2*_*H*_*2*_*-ZF* genes were verified in response to heat, drought, and heat & drought stresses. According to expression pattern analysis, several *TaZFPs*, like *Traes_5BL_D53A846BE.1*, were not only highly expressed in L2DAAs, RTLS, RMS, but also endowed tolerance to drought and heat stresses, making them good candidates for molecular breeding.

**Conclusions:**

This study systematically characterized the TaC_2_H_2_-ZFPs and their potential roles in *T. aestivum*. Our findings provide new insights into the *C*_*2*_*H*_*2*_*-ZF* genes in *T. aestivum* as well as a foundation for further studies on the roles of *TaC*_*2*_*H*_*2*_*-ZF* genes in *T. aestivum* molecular breeding.

**Supplementary Information:**

The online version contains supplementary material available at 10.1186/s12870-021-03016-3.

## Background

The C_2_H_2_-type zinc finger proteins constitute a large family of transcription factors. The C_2_H_2_-ZF domain was defined as about 30 amino acids with two conserved Cysteine and two histidine residues which bound to one Zn^2+^ atom tetrahedrally and form a structure as C-X_2-4_-C-X_3_-F-X_5_-L-X_2_-H-X_3-5_-H [1]. According to the defined C_2_H_2_-ZF types, C_2_H_2_-ZFPs were classified into two subsets X-tandem-SF and X-isolated-SF. The X-tandem-SF contains two clearly subsets (X-t1-SF and X-t2-SF), and the X-isolated-SF contains ten clearly subsets (X-1i-Q-SF, X-1i-M-SF, X-1i-Z-SF, X-1i-D-SF, X-2i-Q-SF, X-2i-M-S and X-2i-Z-SF, X-2i-Mix-SF, X-3i-SF, and X-4i-SF), where X represents any species [2, 3]. Previous studies have shown that *C*_*2*_*H*_*2*_*-ZF* genes represent 0.8%, 2.3%, 3% of all genes in *Saccharomyces cerevisiae*, *Diptera*, *mammals*, respectively [4].

The C_2_H_2_-ZFP family, which have been reported in numerous plant genomes, significantly contribute to the plant growth development, and also participate in multiple biological processes by transcriptional regulation. For instance, genome-wide analyses of ZFP proteins in plants have been carried out in *Arabidopsis* (176), durum wheat (122), rice (189), maize (211), soybean (321), foxtail millet (124) and other species based on genome sequences [2]. In *T. aestivum*, the C1-2i Q-type and C_3_H zinc finger protein (ZFP) transcription factor subclass has been reported to play important roles in plant stress responses and physiological stages [5, 6]. In *Arabidopsis*, the *AZF1*, *AZF2*, *AZF3*, *ZAT6*, *ZAT7*, *ZAT8*, *ZAT10*, *ZAT12* and *ZAT18* have been shown to function in multiple cellular processes, including seed germination, drought, cold, high-salinity, and oxidative stress responses [7–14]. In rice, several members of the C_2_H_2_-type ZFPs, such as *ZFP15*, *ZFP36*, *ZFP39*, *ZFP182*, *ZFP245*, *ZFP252*, and *ZFP179*, have also been shown to be involved in the responses of drought, salinity, and oxidative stress [14–20]. In Poplar (*Populus alba*), *PtrZFP2*/*19*/*95* showed high expression levels in leaves and/or roots under environmental stresses by genome-wide analysis, which provided a solid foundation for studying the biological roles of *C*_*2*_*H*_*2*_*-ZF* genes in Populus growth and development [20].

The conserved amino acid residues of these *C*_*2*_*H*_*2*_*-ZF* genes were consistent with previous studies of the C_2_H_2_ domain in other plant species [10]. These *C*_*2*_*H*_*2*_*-ZF* genes studies include main structural features in C_2_H_2_-ZFPs, such as the arrangement of C_2_H_2_-ZF domains (tandem or dispersed), the length of spacer between the ZFs, the number of C_2_H_2_-ZF domains and the “QALGGH” sequence, whereas yeast and animal C_2_H_2_-ZFPs do not have this motif [3]. Compared with other eukaryote C_2_H_2_-ZFPs, in multiple-fingered C_2_H_2_-ZFPs, the plant zinc-finger domains are separated by long spacers that vary in length and sequence (such as, the double zinc finger protein ZPT2-7 and ZPT2-11 in petunia (*Petumia hybrida*), the distance between its adjacent zinc finger structures is 19 and 65 amino acids, respectively), whereas the C_2_H_2_-ZFPs of yeast and animals are mostly clustered and separated by only six to eight amino acids [4, 21].

Moreover, the structures of C_2_H_2_-ZFPs also contain several non-zinc-finger motifs, such as EAR-motif R-box, PL-box, L-box, and EK-box in *T. aestivum* [22]. The EAR motif (DLNxxP or LxLxL) was showed to be a predominant transcriptional repressor motif in some plant ZFP proteins [22]. Overexpression of most DREB/ERF proteins with an EAR motif led to reduced expression levels of stress-related genes, and fusion of the EAR motif to a number of transcriptional activators was found to convert them to dominant repressors [23]. Thus, the different classifications or different types of C_2_H_2_-ZF domains provides some extrinsic of function information for the *C*_*2*_*H*_*2*_*-ZF* genes, might make C_2_H_2_-ZFP diverse multifunction in comprehensive physiological stages.

Bread wheat (*Triticum aestivum*) as a staple crop of the world plays essential roles to sustain food security [24, 25]. However, dissecting gene function in *T. aestivum* is not easy due to its hexaploid genome and highly redundancy of genes. In this study, the comprehensive analyses were carried out based on phylogenetic relationships, physicochemical properties, subcellular localization prediction, chromosomal locations, and conserved protein domain in bread wheat *C*_*2*_*H*_*2*_*-ZF* genes family. The results of this study will provide the foundation for further functional analyses of the *C*_*2*_*H*_*2*_*-ZF* genes families, important scientific significance and application value for our understanding of the genetics and the evolution of *T. aestivum*.

## Results

### Classification and distribution analysis of *T. aestivum* C_2_H_2_-ZFPs

The 457 putative C_2_H_2_-type Zinc Finger proteins (C_2_H_2_-ZFP) (including 253 splice variants), were identified from the Plant transcription factor database (PlantTFDB) and the iTAK database. Further identification was conducted through the HMM profile HMMER 3.0 program and SMART database search. After removing incomplete and duplicate sequences by using the CD-HIT software, a total of 204 non-redundant candidate genes were obtained to encode 457 proteins, of which 316 proteins in full length and 141 cases as partial in N- and/or C-terminal regions but containing complete C_2_H_2_-ZF domain. In addition, according to the retrieved of the PlantTFDB database, iTAK database and ExPASy server, the 204 T*. aestivum* C_2_H_2_-ZFPs sequences showed that their amino acid length, molecular weight (Mw), isoelectric points (pIs), subcellular localization predictions, for details about TaC_2_H_2_-ZFP, please refer to Additional file 1: Table S2.

**Fig. 1:**
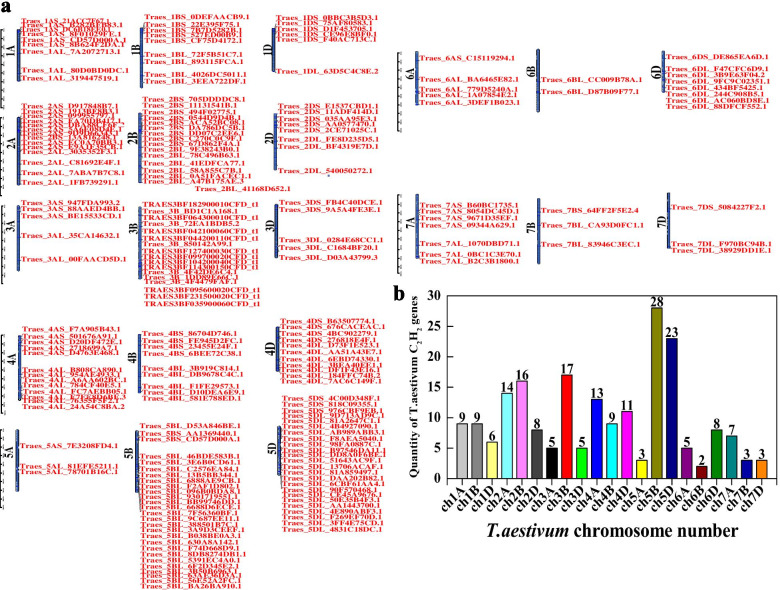
Chromosome localization of TaC_2_H_2_-ZFP members. **a** The TaC_2_H_2_-ZFP members were localized in bread wheat chromosomes (Chr1A, 1B, 1D–7A, 7B, 7D). **b** Numbers of *TaZFP* genes on each *T. aestivum* chromosome

We identified 204 *TaC*_*2*_*H*_*2*_*-ZF* genes in *T. aestivum*, and revealed the distribution of the 204 *TaC*_*2*_*H*_*2*_*-ZF* genes on all the seven chromosomes in both A, B, and D sub-genomes (Additional file 1: Table S2; Fig. 1a). A maximum of 54 (26.5%) *TaC*_*2*_*H*_*2*_*-ZF* genes were located in chromosome 5, 48 on long arms and 6 on short arms, and 5B sub-genomes (26.47%) encoded a maximum number of *TaC*_*2*_*H*_*2*_*-TaZF* genes in the among of all sub-genomes. 38 (18.6%) *TaC*_*2*_*H*_*2*_*-ZF* genes were located in chromosome 2, 14 on long arms and 24 on short arms. The chromosome 7 encoded only 13 *TaC*_*2*_*H*_*2*_*-ZF* genes, and 7 out of 13 were distributed on long arms. 85 (41.67%) *TaC*_*2*_*H*_*2*_*-ZF* genes were encoded in the B sub-genome and 115 (56.37%) *TaC*_*2*_*H*_*2*_*-ZF* genes were distributed on long arms of the chromosomes, suggesting the members of each subsets were not distributed on each chromosome averagely. *TaC*_*2*_*H*_*2*_*-ZF* genes were also more identified on chromosomes 3 and 4, with 27 and 33 genes, respectively (Fig. 1b).

### Phylogeny analysis of C_2_H_2_-ZF domains

Furthermore, according to the variation of the plant-specific conserved amino acid sequence “QALGGH” and distances between metal ligands, the 457 C_2_H_2_-ZF domains of the *T. aestivum* were also classified into four categories (named Q, M, Z and D), the M and Z were further subdivided into M1, M2, M3, and M4, as well as Z1 and Z2 [2]. According to the classification results of the C_2_H_2_-ZF domain, 214 (46.8%) were classified as Ta-tandem-SF and 243 (53.2%) were classified as Ta-isolated-SF. 253 (55%) *TaC*_*2*_*H*_*2*_*-ZF* genes were different splice variants, while contain the same number C_2_H_2_-ZF domains for most *TaC*_*2*_*H*_*2*_*-ZF* genes, only the length of the amino acid sequence is different (Additional file 1: Tables S1 and S2). However, there are some *TaC*_*2*_*H*_*2*_*-ZF* splice variants were exceptions, which number and types of their domains have been reduced and changed. For instance, *Traes_6DL_F47CFC6D9.1*/*2*/*3*/*4*/*5*/*6* have five C_2_H_2_ (M3;M4;M4;M4;Z1) domains, four splice variants *Traes_6DL_F47CFC6D9.7/8*/*9*/*10* have three (M4;M4;Z1), the other three *Traes_6DL_F47CFC6D9.11*/*12*/*13* only have two (M4;Z1) domains. And the C_2_H_2_ domains of *Traes_3AS_BE15533CD.1*/2 (Z1;D)-C_2_H_2_ domains are completely different from its variant *Traes_3AS_BE15533CD.3* (M3-C_2_H_2_ domain). Undoubtedly, different splice variants of a *TaC*_*2*_*H*_*2*_*-ZF* gene have different number or type C_2_H_2_ domains, may perform different functions [26]. For the detailed information of the classified C_2_H_2_-ZFPs in *T. aestivum*, see Additional file 1: Table S1.

**Fig. 2:**
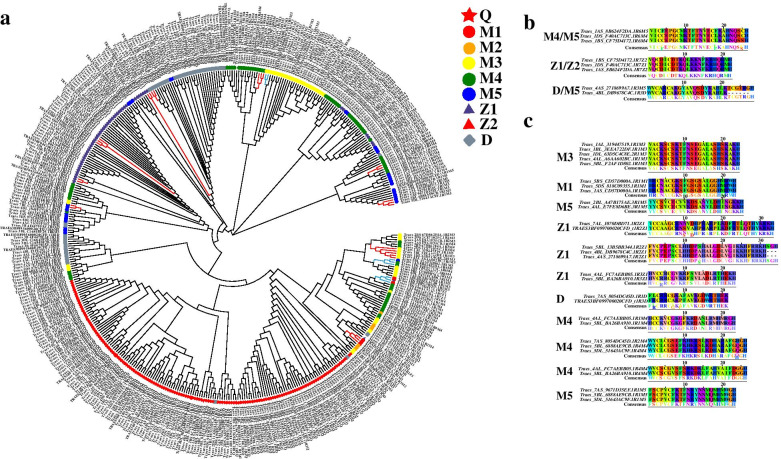
Phylogenetic tree showing the spread of C_2_H_2_ domain proteins of *T. aestivum*. The phylogenetic tree was constructed using the neighbor-joining method as implemented in MEGA 7.0 from TaC_2_H_2_-ZF protein sequences alignment. Bootstrap values from 1000 replicates are displayed at each node. The different C_2_H_2_ domain proteins on the tree can be divided by different colors

To evaluate the evolutionary relationships of the *C*_*2*_*H*_*2*_ gene family in *T. aestivum*, an unrooted phylogenetic tree was constructed with the neighbor-joining method based on the domain sequence alignment of 527 conserved ZFP domains (Fig. 2a). Through the analysis of domain sequence alignments, our analysis showed that most members were well separated. The majority of C_2_H_2_-ZF proteins in the same clade, particularly the most closely related proteins, typically shared common types (for example, Q-type, M1-type), indicating a similar potential function between these C_2_H_2_-ZF proteins. Within each subfamily member in a branch, the strong amino acid sequence conservation is evident from the short branch lengths at the tips of the trees, suggestion of strong evolutionary relationships among subfamily members (Fig. 2c). However, there are clear exceptions, and the different type subfamilies of C_2_H_2_-ZF proteins are found in the same clade (Fig. 2b, c). Our results cast a new light on classification of C_2_H_2_-ZF domain.

### Gene structure and conserved motif analysis of C_2_H_2_-ZFPs

**Fig. 3:**
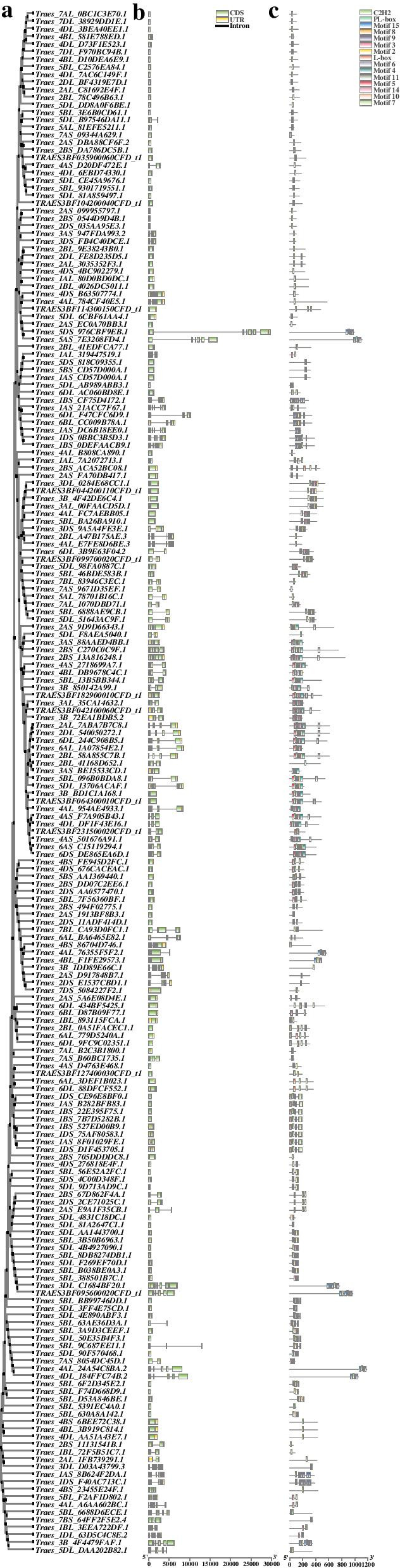
Gene structure and motifs of *TaC*_*2*_*H*_*2*_*-ZFP* family. **a** The clustering of TaC_2_H_2_-ZF proteins based on Maximun Likelihoodphy phylogenetic tree. **b** The exon–intron structure of *TaC*_*2*_*H*_*2*_*-ZF* genes, the yellow boxes are the UTR region of *TaC*_*2*_*H*_*2*_*-ZF* genes, the green boxes are the exon of CDS, and the black line is the intron. **c** Schematic depiction of 15 conserved motifs in TaC_2_H_2_-ZF proteins. MEME online tool was used to identify the motifs of the TaC_2_H_2_-ZF proteins. Each motif is denoted by different colored blocks with respective numbers in the center of the motifs. The number in boxes (1–15) signifies motif 1–motif 15. The length and position of each colored box indicate the actual size of the motif. The full size and high resolution figure is available in Additional file 2

To further explore the evolutionary relationship among *TaC*_*2*_*H*_*2*_*-ZF* genes, an unrooted neighbor-joining phylogenetic tree of 204 TaC_2_H_2_-ZFPs full-length amino acid sequence was constructed. The gene structures and motif characteristics of TaC_2_H_2_-ZFPs were analyzed (Fig. 3a). The results of Gene Structure Display Server (GSDS) analysis showed that the number of introns in the *TaC*_*2*_*H*_*2*_*-ZF* genes contained from zero to ten introns, most of the genes had one to three introns (104 genes have no introns) (Fig. 3b), and most members contained typical C_2_H_2_ domains. The lengths of individual TaC_2_H_2_-ZFP were variable in intron length and it could partly reflect the length of different genes. For instance, the longest gene, *Traes_5DS_976CBF9EB.1*, with a size of 29.1 kb, was due mainly to the fact that it contained a longest intron with length of 21.9 kb.

To identify common motifs among different groups of TaZFPs, we employed the MEME Version 5.1.1 [27] software to analyze the conserved motifs in amino acid sequences of 204 *TaC*_*2*_*H*_*2*_*-ZFP* genes in *T. aestivum*. Figure 3c shows the symbols of the motifs and the composition of the 204 *TaC*_*2*_*H*_*2*_*-ZFP* in the C_2_H_2_ family. Protein domain analysis showed that most members contained typical C_2_H_2_ domains. The C_2_H_2_ domains were annotated as the ZFP specific domains, in their N-terminal and C-terminal regions. Fifteen specific motifs were defined, with the ZFPs of subset Ta-t1-SF (*Traes_6DL_3B9E63F04.2*) containing the largest number of motifs. The TaC_2_H_2_-ZFPs belonging to the same subset have a similar motif composition (e.g. *Traes_7AL_0BC1C3E70.1* and *7DL_38929DD1E.1*, *1AS_21ACC7F67.1* and *6DL_F47CFC6D9.1*). Also, some motifs appeared in only certain specific subsets. For example, motif 5 was unique to subset Ta-t1-SF and Ta-1i-M-SF, while L-box was specific to subset Ta-2i-Q-SF and Ta-3i-SF. However, it may be due to the divergence of the C_2_H_2_ motif, especially the diverse motif numbers and spacing in the amino acid sequences between Cys and Cys or Cys and His in each protein. Overall, aboved analysis suggested that the C_2_H_2_-ZFPs with only one and two Q-type C_2_H_2_ domains were most conservative in *T. aestivum*.

### Discovery of conserved motifs specific of the C_2_H_2_-type zinc finger domain: SFP1-, DL-, R-, PL-, L-, EK-, and NPL-box

**Fig. 4:**
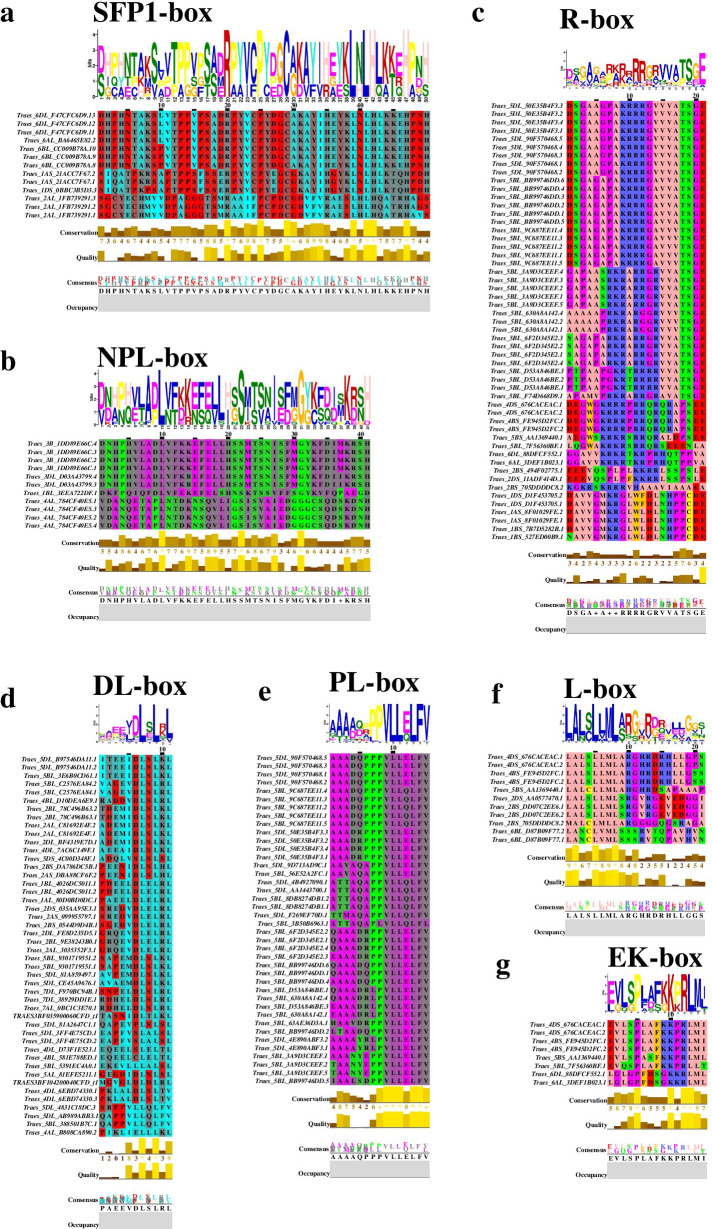
The conserved functional motifs and sequence logos of ten subsets proteins were predicted by MEME software. The highlights show the *T. aestivum* genome conservation of the C_2_H_2_ zinc finger motif. Numbers on the x-axis represent the sequence positions in zinc finger motifs. The y-axis represents the information content measured in bits

Several other conserved motifs except C_2_H_2_ subfamily members were detected by MEME algorithm and multiple sequence alignments (Fig. 4a-g). The C_2_H_2_ subset Ta-1i-M-SF is composed of ZF proteins that contain not only the C_2_H_2_-ZF domain but also a Nucleoplasmin-like (NPL) domain [28, 29]. Ta-2i-Mix-SF contains Schizosaccharomyces pombe finger protein 1 (SFP1) domain [30, 31]. Ta-1i-Q-SF contains DL-box. Ta-2i-Q-SF contain R-box, PL-box, L-box, and EK-box, respectively [23] (Fig. 4c-g). NPL-box (Ta-1i-M-SF) and L-box (Ta-2i-Q-SF) are located in the N-terminal region of C_2_H_2_-ZF domains. DL-box and R-box (Ta-1i-Q-SF) are located in the C-terminal region of C_2_H_2_-ZF domains. SFP1-box and R-box (Ta-2i-Q-SF) are located in the C-terminal region of the first C_2_H_2_-ZF domains. PL-box (Ta-2i-Q-SF) and EK-box are located in the C-terminal region of the second C_2_H_2_-ZF domains. Conservation motif of L-box, NPL-box and SFP1-box in some C_2_H_2_-type zinc fingered proteins have been showed in the previous studies [29, 31].

The DL-box is similar to the previously reported EAR motif (LDLSL) and may make the proteins contain transcriptional repressor functions [32]. Another promising finding was that different splice variants of many genes contained different motifs. For instance, two splice variants of Traes_5DL_50E35B4F3 contain R-box and PL-box, respectively. Traes_4DS_676CACEAC contain R-box, L-box, and EK-box in three splice variants. It indicated that the different subset members maybe contain a novel motif and execute different biological functions.

### The C_2_H_2_ family genes in *T. aestivum* were more closely evolutionary relationship with *Poaceae*

**Fig. 5:**
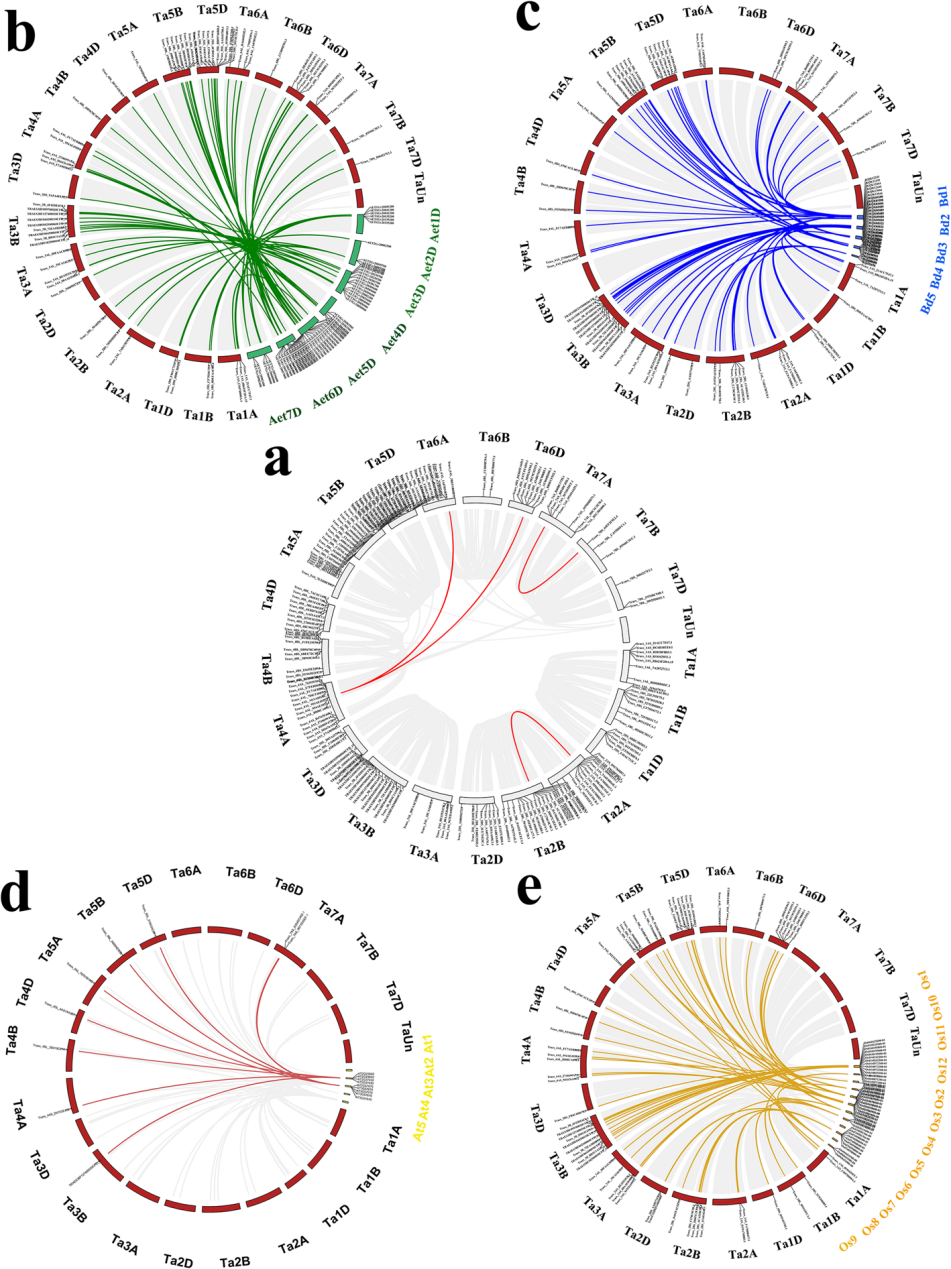
The collinearity analysis of the *C*_*2*_*H*_*2*_*-ZF* gene family in *T. aestivum*. **a** Colinearity analysis of *TaZFP* genes. Duplicated gene pairs in the *T. aestivum* genome. **b** colinearity analysis of *TaZFP* genes with *Aegilops tauschii*. **c ***Brachypodium distachyon*. **d ***Arabidopsis thaliana*. **e ***Oryza sativa Japonica* (rice). Gray lines in the background indicate the collinear blocks within the *T. aestivum* and other plant genomes, while the dark lines highlight the syntenic *TaZFP* gene pairs

To further infer the phylogenetic mechanisms of the *T. aestivum C*_*2*_*H*_*2*_*-ZF* gene family and to better understand the origin, we constructed five comparative syntenic maps of *T. aestivum* associated with four representative species, including three monocots (*A. tauschii*, *B. distachyon*, and rice) and one dicot (*Arabidopsis*) (Fig. 5a-e). Only four pairs of C_2_H_2_ family genes in *T. aestivum* showed syntenic relationships. In four representative species, there was a syntenic relationship between C_2_H_2_ family genes and 65 genes in *B. distachyon*, 66 genes in *A. tauschii*, 68 genes in rice, 9 genes in *Arabidopsis*, which indicated that C_2_H_2_ family genes in *T. aestivum* were closely related to genes in the *Poaceae* family. More importantly, we found that some *C*_*2*_*H*_*2*_*-ZF* genes were associated with more gene pairs, such as *Traes_2DS_2CE71025C.1* of the *C*_*2*_*H*_*2*_ gene family in *T. aestivum* was the syntenic relationship with *KQK10684* and *KQK06003*. The *Os05t0114400-01* is syntenic relationship with *Traes_4AL_E7FE8D6BE.3*, *7BL_83946C3EC.1* and *1BS_527ED00B9.1*. The results indicated that these genes might have similar functions. Furthermore, there was a high degree of syntenic blocks among the *T. aestivum*, *A. tauschii*, *B. distachyon*, and rice as shown in Fig. 5a-e. In contrast, fewer gene pairs were located in syntenic blocks between *T. aestivum* and *Arabidopsis*, which may be related to the phylogenetic relationship between *T. aestivum* and three plant species.

Interestingly, 33 *TaC*_*2*_*H*_*2*_*-ZF* genes in *T. aestivum* were found to be collinear with *ZFP* gene in *A. tauschii*, as well as with ZFP gene in *B. distachyon* and rice, but not found between *T. aestivum* and *Arabidopsis*, such as *Traes_6DL_88DFCF552.1*/*KQK08112* and *4AL_24A54C8BA.2*/*Os05t0106000-01*, which indicated that these orthologous pairs formed after the divergence of dicotyledonous and monocotyledonous plants. Additionally, one *TaC*_*2*_*H*_*2*_*-ZF* gene in *T. aestivum* was identified to be collinear with *ZFP* genes in *A. tauschii*, *B. distachyon*, rice, and *Arabidopsis*, indicating that these orthologous pairs may already exist before the ancestral divergence (Additional file 1: Table S3; Fig. 5a-e).

To better understand the evolutionary constraints acting on this gene family, we calculated the Ka/Ks ratios for the *TaC*_*2*_*H*_*2*_*-ZF* gene pairs. Generally, Ka/Ks > 1 indicates positive selection, Ka/Ks = 1 indicates neutral selection, and Ka/Ks < 1 indicates purification [33]. Additional file 1: Table S3 showed that the all Ka/Ks ratios were less than 1, suggesting that *TaC*_*2*_*H*_*2*_*-ZF* genes were undergone purifying selection, and it was speculated that their gene functions did not differentiate, which largely maintained the functional similarity of the members of the TaC_2_H_2_-ZFP family. The divergence time of duplication events were inferred by Ks (Additional file 1: Table S3). We predicted that the divergence time of *C*_*2*_*H*_*2*_*-ZF* gene pairs should be approximately 286 Mya (*T. aestivum*), 20 Mya (*A. tauschii*), 37 Mya (*B. distachyon*), 39 Mya (rice), 64 Mya (*Arabidopsis*) (Additional file 1: Table S3). The result indicated that *TaC*_*2*_*H*_*2*_*-ZF* gene family shared an intimate correlation with those in *A. tauschii*, *B. distachyon*, rice, and *Arabidopsis*.

### Expression profiles of *T. aestivum TaC*_*2*_*H*_*2*_*-ZF* genes

To explore the expression patterns of *T. aestivum C*_*2*_*H*_*2*_*-ZF* genes during development and in response to abiotic stresses, we used publicly available RNA-seq data and mapped to the bread wheat genome by Borrill [34]. These samples included diverse developmental stages, tissues, and abiotic stress conditions. The RNA-seq data corresponded to 5 organs at 15 developmental stages [35].

 To detect preferentially expressed *TaC*_*2*_*H*_*2*_*-ZF* genes in certain *T. aestivum* tissues and at certain stages, we found that totally 198 of 204 *TaC*_*2*_*H*_*2*_*-ZF* genes were expressed in all organs and development stages, and 37 of 204 *TaC*_*2*_*H*_*2*_*-ZF* genes were responded to heat, drought, and heat & drought abiotic stresses according to public transcriptome data.

**Fig. 6:**
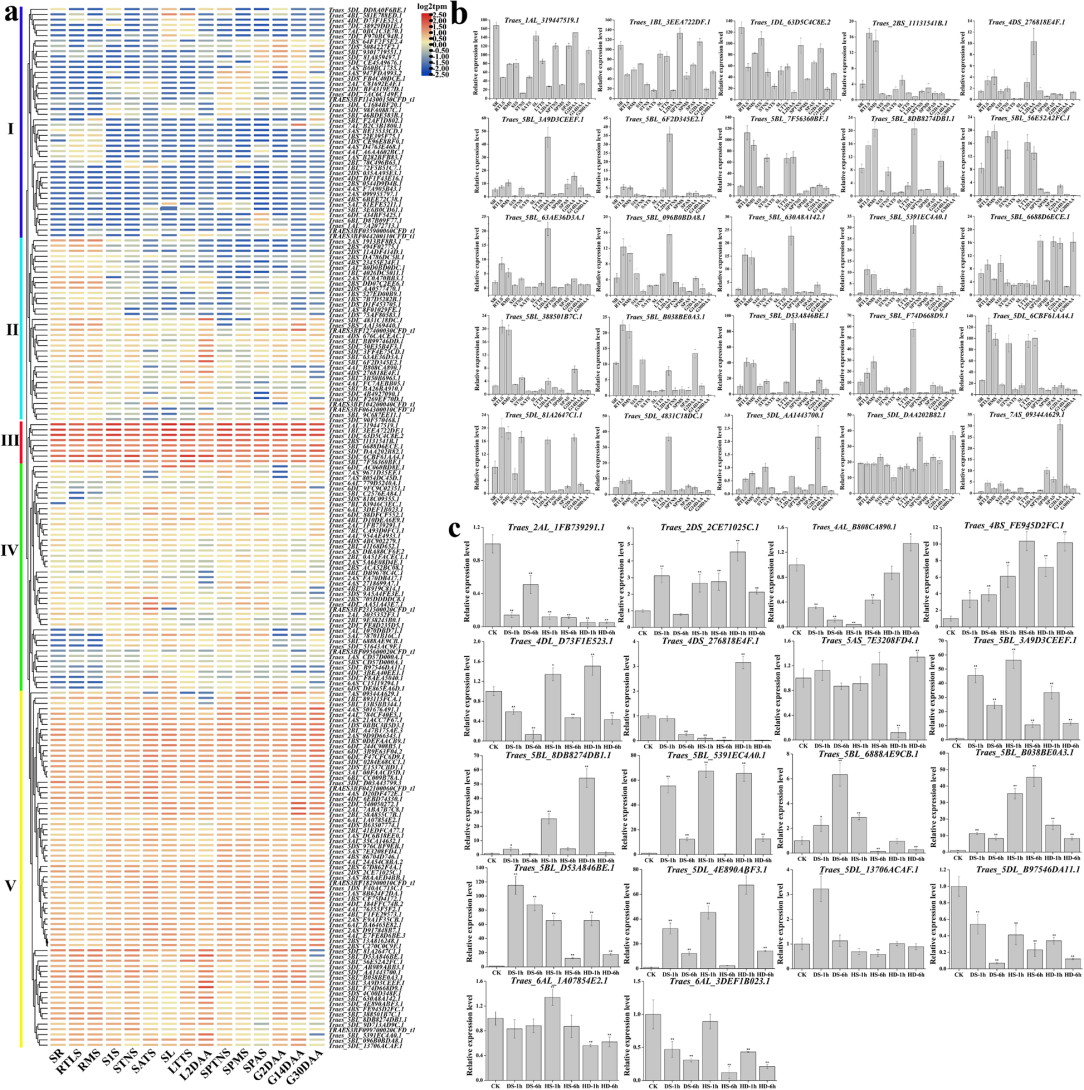
Transcriptome analysis of *T. aestivum C*_*2*_*H*_*2*_*-ZF* genes. **a** Expression profiles of 198 *C*_*2*_*H*_*2*_*-ZF* genes in different *T. aestivum* tissues. Hierarchical clustering of the relative transcript abundance profiles (log2 tpm) of genes. Different colors degrees represent different expression level. The scale bar indicates relative expression level as shown on the top right side. The different tissue types are shown on the bottom side: seedling roots (SR), roots at the three leaves (RTLS), roots at the meiosis (RMS), stems at the 1 cm spike (S1S), stems at the two nodes stems (STNS), stems at the anthesis stems (SATS), seedling leafs (SL), leafs at the three tillers (LTTS), leafs at the 2 days after anthesis (L2DAAs), spikes at the two nodes stems (SPTNS), spikes at the meiosis (SPMS), spikes at the anthesis stems (SPAS), grains at the 2 days after anthesis (G2DAAs), grains at the 14 days after anthesis (G14DAAs), grains at the 30 days after anthesis (G30DAAs). The individual gene names are indicated on the right side. **b** Expression analysis of 25 *TaC*_*2*_*H*_*2*_*-ZF* genes in different tissues and developmental stages by qRT-PCR, vertical bars indicate standard deviation. **c** Expression analysis of 18 *TaC*_*2*_*H*_*2*_*-ZF* genes in response to drought and heat stress by qRT-PCR, and vertical bars indicate standard deviation. The full size and high resolution figure is available in Additional file 3

A heatmap showed the relative expression profile of 198 *TaC*_*2*_*H*_*2*_*-ZF* genes in 5 different organs and 15 different development stages (Additional file 1: Table S5; Fig. 6a). The 204 *TaC*_*2*_*H*_*2*_*-ZF* genes were clustered into five groups. The 44 genes (22.2%) of group I were lowly transcript accumulated in most organs and development stages. The 35 genes (17.7%) of group II were all preferentially moderately expression in the root. Among 35 genes, the 9 genes (25.7%) were moderately expression in stem, leaf, spike and grain, and 26 genes (74.3%) were lowly transcript accumulation in stem, leaf, spike and grain. The 8 genes (4.1%) of group III were highly transcript accumulation in all organs and development stages. The 43 genes (21.7%) of group IV were moderately expressed in most organs and development stages, but in which 12 genes (27.9%) were lowly transcript accumulation in root. The 74 genes (37.4%) of group V were moderately and consecutively expressed in all organs and development stages (Fig. 6a). We found that the Ta-t1-SF members were mainly distributed into the expression group V (44.6%), while the Ta-1i-Q-SF into group I (54.5%), the Ta-2i-Q-SF into group II (60%), the Ta-1i-M-SF into group III (50%) and IV (27.9%).

To verify the expression profiles of *TaC*_*2*_*H*_*2*_*-ZF* genes obtained by the RNA-seq data analysis, qRT-PCR was performed for 43 selected genes (25 *TaC*_*2*_*H*_*2*_*-ZF* genes that showed a large change in different tissues expression, 18 *TaC*_*2*_*H*_*2*_*-ZF* genes is very significantly in response to drought and heat stresses). The gene-specific primers are listed in Additional file 1: Table S4. As shown in Fig. 6b and c, among the 25 genes, 3 genes showed the highest transcript accumulation in all development stages. 4 genes in the root (seedling, three leaves, and meiosis), stem (Spike at 1 cm and two nodes), leaf (seedling, Three tillers), grain (2 DAAs and 30 DAAs). The one genes showed moderately transcript accumulation in the root (three leaves and meiosis), stem (two nodes), leaf (Three tillers and 2 DAAs). The one gene showed moderately transcript accumulation in the root (three leaves and meiosis), leaf (2 DAAs). The 15 genes showed moderately transcript accumulation in the root (three leaves and meiosis), leaf (2 DAAs), spike (two nodes, meiosis, and anthesis), grain (2 DAAs and 30 DAAs), respectively. These results were almost consistent with those observed in the RNA-seq data analysis, while the levels of upregulation were slightly different (Fig. 6a, b). The expression of *Traes_5DL_AA1443700.1* has almost no change at all stages, which is slightly different from the results of the above-mentioned RNA-seq data analysis (Fig. 6b).

Subsequently, the expression profiles of the 18 *TaC*_*2*_*H*_*2*_*-ZF* genes were further analyzed under drought and heat stresses, and most of which were well following microarray profiles. The qRT-PCR results were highly consistent with the RNA-seq data. Among the 18 *TaZFPs*, *Traes_2AL_1FB739291.1* and *5DL_B97546DA11.1* were observed to be increased in response to drought and heat stress. The *4DS_276818E4F.1* was upregulated significantly at HD-1 h. The *4AL_B808CA890.1* was suppressed after DS-1 h, DS-6 h and HS-1 h treatments, whereas it was upregulated after HS-6 h, HD-1 h, and HD-6 h treatment (Fig. 6c). Notably, *Traes_5BL_B038BE0A3.1*, *5BL_3A9D3CEEF.1* and *5BL_D53A846BE.1* displayed significantly high expression levels at all treatment time points. The *5BL_5391EC4A0.1* and *5DL_4E890ABF3.1* displayed significantly high expression levels at DS-1 h, DS-6 h, HS-1 h, HD-1 h and HD-6 h treatment time points.

In summary, our results showed that these selected *TaC*_*2*_*H*_*2*_*-ZF* genes responded to one or more stresses. According to expression pattern analysis found, like *Traes_5BL_D53A846BE.1*, not only expressed highly in L2DAAs, RTLS, RMS, but also endowed tolerance to drought and heat stresses, making them good candidates for molecular breeding.

## Discussion

### *T. aestivum* C_2_H_2_-ZF proteins have a relatively conservative evolutionary process

C_2_H_2_-type zinc finger proteins belongs to one of the largest plant TF families and participates in multiple biological processes. Therefore, *T. aestivum C*_*2*_*H*_*2*_*-ZF* genes may be promising targets for crop breeding and improvement.

**Fig. 7:**
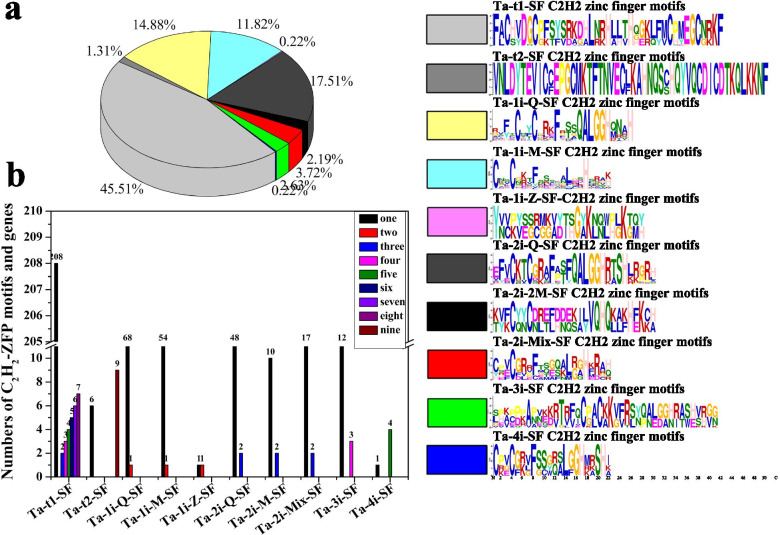
Subset classification of *C*_*2*_*H*_*2*_*-ZF* genes in the *T. aestivum* genome. **a** Numbers of C_2_H_2_-ZFPs in the 10 different subsets. **b** Numbers of C_2_H_2_-ZFPs containing 1-7 and 9 C_2_H_2_-ZF domains

The 457 putative C_2_H_2_-type Zinc Finger proteins (C_2_H_2_-ZFP) (including 253 splice variants) were identified from *T. aestivum.* There are 179, 122, 189, 321 (including 135 pairs and 22 single genes), and 124 C_2_H_2_-ZFP members in *Arabidopsis*, durum wheat, rice, soybean, foxtail millet, respectively [1, 2] (Fig. 7a, b). Compared with the number of above species C_2_H_2_-ZFPs family members, *T. aestivum* have one main features in most of the plant C_2_H_2_-ZFPs due to its hexaploidy genome probably. in this study, *T. aestivum* C_2_H_2_-ZFPs were classified into four great categories (named Q, M, Z, and D) according to the variation of the conserved amino acid sequence “QALGGH” and distances between metal ligands, and 457 C_2_H_2_-ZFPs were further subdivided into ten different subsets, based on the arrangements, numbers, and types of C_2_H_2_-ZF domains [1] (Additional file 1: Table S1; Figs. 2a and 7a-b). This result was well identified by phylogenetic analysis (Fig. 2a). Previous studied have demonstrated that the molecular functions of the C_2_H_2_-ZFPs with tandem C_2_H_2_-ZF domain are generally different from the C_2_H_2_-ZFPs with dispersed fingers [36–38]. So, in this study, the TaC_2_H_2_-ZFPs with single or dispersed C_2_H_2_-ZF domains are generally classified separately from tandem C_2_H_2_-ZFP domain proteins. The TaC_2_H_2_-ZFPs were classified into two Ta-tandem-SF subsets and eight Ta-isolated-SF subsets (Additional file 1: Table S1; Fig. 7a, b). According on the statistical analysis results of linker lengths in *T. aestivum* C_2_H_2_-ZFPs, Ta-tandem-SF is different from the tandem ZFs in yeast and *Arabidopsis* (Additional file 1: Table S1) [36, 39]. Two Ta-tandem-SF subsets and eight Ta-isolated-SF were defined based on the numbers of C_2_H_2_-ZF domains according to the tandem-SF and isolated-SF in soybean and *Brassica rapa* L C_2_H_2_-ZFPs [2, 3]. In addition, two Ta-tandem-SF had no Q-type C_2_H_2_-ZF domain in these C_2_H_2_-ZFPs, which was similar to the yeast and animals [36]. Furthermore, the Q-type and M-type C_2_H_2_-ZF domains were similar, Z-type and D-type were completely different from the types (Additional file 1: Table S1), as these were defined in previous studies [2, 3, 40, 41].

We also examine the C_2_H_2_-ZF domains type contained in the 11 subsets, and found that the Ta-1i-Q-SF, Ta-2i-Q-SF, Ta-2i-Mix-SF, Ta-3i-SF and Ta-4i-SF subsets of *T. aestivum* C_2_H_2_-ZFPs members contain one or two the QALGGH motif, and were identified as Q-type C_2_H_2_-ZFPs. (Additional file 1: Table S1; Fig. 7) [42]. However, the Q-type C_2_H_2_-ZFPs is considered as plant specific [43], animal and yeast lack this motif, which suggesting that Q-type C_2_H_2_-ZFPs might have evolved for as specific regulatory process unique in plants. And in the current study, the D, M1, M2, M3, M4, and M5 domains are generally generated is due to the simultaneous mutation of any first five amino acids in the conserved sequence QALGGH, respectively, leading to the different protein functions of TaC_2_H_2_-ZFPs [2, 3]. The complexity of these C_2_H_2_-ZF domains as well as the presence of other known domains in some C_2_H_2_-ZFPs indicates the functional diversity of these C_2_H_2_-ZFPs in plant growth and development. Our classifications of *T. aestivum* C_2_H_2_-ZFPs provided helpful information for further functional characterization of C_2_H_2_-ZFPs gene family among *Poaceae*.

In the current study, our analysis found that C_2_H_2_-ZFPs family genes in *T. aestivum* were closely related to genes in the *Poaceae* family. Most *TaC*_*2*_*H*_*2*_*-ZF* genes have orthologous genes in *A. tauschii*, *B. distachyon*, rice, and *Arabidopsis* (Fig. 4b-e, Additional file 1: Table S3), there are 66, 65, 68, and 9 *C*_*2*_*H*_*2*_*-ZF* gene pairs between *T. aestivum* and *A. tauschii*, *B. distachyon*, rice, and *Arabidopsis*, respectively. And they are 7.2 times, 7.4 times, and 7.6 times of number of the gene pairs between *T. aestivum* and *Arabidopsis*, respectively. From the results of gene replication, 33 *TaC*_*2*_*H*_*2*_*-ZF* gene pairs were duplicated genes and the high levels of collinear gene pairs were observed among *A. tauschi*, *B. distachyon*, and rice. The number of segmental duplication events was greater than that of tandem duplication events, indicating that segmental duplication contributed to *TaC*_*2*_*H*_*2*_*-ZF* genes expansion.

The analysis of syntenic and Ka/Ks values indicated that no positive selection occurred and underwent strong purifying selection in *TaC*_*2*_*H*_*2*_*-ZF* genes, and the results were consistent with the studies of *C*_*2*_*H*_*2*_*-ZF* genes in other plants [2, 3]. Overall, we inferred that the evolution of the *TaC*_*2*_*H*_*2*_*-ZF* gene family was similar to that in other plants. Compared with *Arabidopsis*, *T. aestivum C*_*2*_*H*_*2*_-ZF genes shared a strong relationship evolutionary with *A. tauschi*, *B. distachyon*, and rice *C*_*2*_*H*_*2*_*-ZF* genes.

### Subfamily specific motifs (SFP1-, DL-, R-, PL-, L-, EK-, and NPL-box) of *T. aestivum C*_*2*_*H*_*2*_*-ZF *genes may contribute to the high adaptability of *T. aestivum*

Subsequently, sequence logo analysis to explore the sequence characteristics of the C_2_H_2_ domains from the ten major subsets were studied by the MEME motif search tool. The TaC_2_H_2_-ZF domains not only contain the C_2_H_2_ domain but also contain NPL, SFP1, DL-box, R-box, PL-box, L-box, and EK-box [29–32] (Fig. 4a-g). These motifs were previously reported in rice and *Arabidopsis*, [22].

The L-box have been shown in some 2-fingered Q-type ZFP proteins, such as AZF2 (*Arabidopsis*), ZFP15 (rice) and ZFP36 (rice) [19, 44, 45]. In the present study, all TaC_2_H_2_-ZFPs containing L-box also contained 2-fingered Q-type C_2_H_2_-ZF domain and belonged to Ta-2i-Q-SF subsets, suggesting L-box has a certain function with two Q-type ZF domain. For instance, *Traes_6BL_D87B09F77.1* was homologous to rice *ZFP15*, both containing 2 Q-type C_2_H_2_-ZF domains and a L-box each. *Traes_6BL_D87B09F77.1* expressed highest in the SPAS stages than other developmental stages. Meanwhile, transgenic rice *ZFP15* showed it was important for pike development [45]. All these results suggested *Traes_6BL_D87B09F77.1* has a certain role in spike development.

The DL-box is similar to the previously reported LDLNL-EAR motif suggesting it may function as transcriptional repressors [32]. Previous study showed that the EAR motif could reduce the transcriptional level of the reporter gene, and the transcriptional activation activity of some TFs, as well as negatively regulated genes involved in developmental, hormonal, and stress signaling pathways [46, 47]. For instance, overexpression of intact DREB/ERF proteins with an EAR motif led to the reduced expression levels of stress-related genes and decreased abiotic stress tolerance [23, 48, 49]. Furthermore, when EAR motif was tethered to transcriptional activators, the EAR motif functioned as dominant repressors [50]. In this study, we found that Traes_4AL_B808CA890.1, Traes_4DL_D73F1E523.1, and Traes_5DL_B97546DA11.1, all belonged to Ta-1i-Q-SF,contain LDLNL-EAR-motif-like domain. And through existing transcriptome data and real-time quantitative data analysis, it was found that the transcriptional levels of these three genes were decreased under drought and heat stress conditions. If a mutation occurs in this DLDLNL inhibitory domain, the protein's inhibitory function is abolished, For example, the EAR domain of TaRAP2.1L is responsible for a negative effect on growth and stress tolerance, while it became an activator after the EAR-motif was mutated to xAAAxxA from xDLNxxP [23, 51]. In briefly, both negative and/or positive regulators of gene expression containing the EAR motif domain play key roles in the regulation of plant stress responses.

The above analysis showed these TaC_2_H_2_-ZFPs had the high functional diversity in plant growth and development. This study cast a new light on multifunction of *TaC*_*2*_*H*_*2*_*-ZF* genes, and provided a certain reference value for people to understand the functions of the *C*_*2*_*H*_*2*_*-ZF* genes in *T. aestivum*. It is also worth discussing these significant facts revealed by the results of the above analysis.

### Expression profiles revealed that TaZFPs comprehensively involved in development and abiotic stress responses

The RNA-seq analysis showed that 198 of 204 T*. aestivum C*_*2*_*H*_*2*_*-ZF* genes were expressed in 5 organs at 15 developmental stages. 134 (67.7%) of the *TaC*_*2*_*H*_*2*_*-ZF* genes were moderately and consecutively expressed in root and leaf (Fig. 6a). In plants, roots and leaves are the most important organs necessary for water and nutrient absorption, also crucial for water content regulation [52]. *T. aestivum* is widespread over the dryland world, and is grown in low rainfall condition, so drought/heat stress response are important for *T. aestivum* [48, 49]. Since dominant *TaC*_*2*_*H*_*2*_*-ZFPs* expressed in root and leaf, TaC_2_H_2_-ZFP transcription factors will is a major target for *T. aestivum* study breeding against drought stress mechanism. The study also indicated that *T. aestivum* C_2_H_2_-ZFPs played roles in root and leaf development more than other tissues or organs. There is considerable evidence that C_2_H_2_-ZFP transcription factors are involved widely in the development of multiple organs and tissues and abiotic stresses, such as seed maturation, floral development, spike development [1], salt stress tolerance [15, 53], drought stress tolerance [54].

In this study, we examined the expression patterns of 43 selected genes. The *TaC*_*2*_*H*_*2*_*-ZFP* genes of chromosome 5 account for 67.4% (Fig. 6a). Previous studies have shown that some *C*_*2*_*H*_*2*_*-ZF* genes located in chromosome 5 play important roles in the *T. aestivum* growth development stage and abiotic stress processes, such as *WZF1*, *TaZFP15*, *TaZFP34* [55–57]. And most results were broadly consistent with the microarray profiles by real-time quantitative RT-PCR verification.

The *Traes_5DL_6CBF61AA4.1* and *5BL_7F56360BF.1* were homologous to *Arabidopsis AtZAT10* (*AT1G27730.1*, also called *STZ*). The three genes belong to the same subsets and all of them contain not only the Q-type C_2_H_2_-ZF domain but also a FDLNI (EAR-like motif) and an EK-box. *Traes_5DL_6CBF61AA4.1* and *5BL_7F56360BF.1* were highly expressed in the SR, RTLS, RMS, S1S, STNS, LTTS and L2DAAs stages. Moreover, transgenic *Arabidopsis* overexpressing *AtZAT10* showed growth retardation as demonstrated in a previous study [7, 22], suggesting that the functions of three homologous genes were probably conserved in both species.

Similarly, the *Traes_4BS_FE945D2FC.1*, homologous to *Arabidopsis AT3G19580.2* (*AZF2*)*,* contains 2 Q-type C_2_H_2_-ZF domains, a FDLNI (EAR-like-motif), an EK-box, and a L-box. Previous analysis showed that expression of *AZF2* was strongly induced by drought stresses [7, 22]. The *Traes_4BS_FE945D2FC.1* was significantly up-regulated at 6 h (up to tenfold) under heat & drought stresses, suggesting it had a similar function in drought stresses with *AZF2*. fThe *Traes_5BL_D53A846BE.1* highly homologous to *Arabidopsis AT2G37430.1* (*ZAT11*) and *AT5G59820* (*ZAT12*), contains 2 Q-type C_2_H_2_-ZF domains. The *Traes_5BL_D53A846BE.1*, not only highly expressed in the RTLS, RMS and L2DAAs stages, but also responded to drought and heat stresses, making them good candidates for molecular breeding. While the *ZAT11* is highly expressed in roots and particularly in root tips [58]*.* The *ZAT12* can reduce heat-derived oxidative stress by activating multiple defense genes in tomato [59]. These results indicated that all the tested genes were more or less involved in development stages and stress response in *T. aestivum*, in a similar way as their orthologs in *Arabidopsis*. Further tissue expression and stress responsive expression analysis of a larger number of *T. aestivum C*_*2*_*H*_*2*_*-ZF* genes, including those lowly expressed genes, would allow to identify the most actives members in gramineae crops.

The above results indicated that these *C*_*2*_*H*_*2*_*-ZF* genes might be involved in the important regulatory networks or cross-talk triggered by different responses stresses. Overall, these findings provide insight into the potential functional roles of *T. aestivum* *C*_*2*_*H*_*2*_*-ZF* genes. The results provide a basis for further functional study of *C*_*2*_*H*_*2*_*-ZF* genes.

## Conclusions

In the present study, we systematically analyzed the characterization of the TaZFP family. The 457 *C*_*2*_*H*_*2*_*-ZF* genes in the *T. aestivum* genome were identified (including 253 splice variants), according to defined C_2_H_2_-ZF domain types, and C_2_H_2_-ZFPs were classified into 10 distinct subsets. A total of 204 non-redundant candidate genes were obtained to encode 457 proteins. The 85 genes (41.67%) were encoded on the B sub-genome and 115 genes (56.37%) were distributed on long arms of the chromosomes. Synteny analysis showed that only 4 pairs of C_2_H_2_ family genes in *T. aestivum*, 65 genes in *B. distachyon*, 66 genes in *A. tauschii*, 68 genes in rice, 9 genes in *Arabidopsis*, were syntenic relationships respectively, indicating that TaZFPs were closely related to genes in the *Poaceae*. The specific conserved motifs of the C_2_H_2_-type zinc finger domain including SFP1-, DL- (EAR-like-motif), R-, PL-, L-, EK- and NPL-box were discovered, indicating the different subset members maybe contain a novel motif and execute different biological functions. According to public transcriptome data, totally 198 of 204 *TaC*_*2*_*H*_*2*_*-ZF* genes were found to have the expression data. The 25 *TaC*_*2*_*H*_*2*_*-ZF* genes were certificated to be significantly differentially expressed in 5 different organs and 15 different development stages by quantitative RT-PCR. The 18 *TaC*_*2*_*H*_*2*_*-ZF* genes were verified in response to heat, drought, and heat & drought stresses. Such as *Traes_5BL_D53A846BE.1*, it was not only highly expressed in L2DAAs, RTLS and RMS, but also endowed tolerance to drought and heat stresses, making them good candidates for molecular breeding. In summary, the present study provides the valuable reference information for further study of *C*_*2*_*H*_*2*_*-ZF* genes, and provides promising targets for further genetic engineering and genetic improvement in *T. Aestivum*.

## Methods

### Plant materials and abiotic stress treatments

Bread wheat (*Triticum aestivum* L. cv. Fielder) materials were acquired from Prof. Xue Gangping's lab in CSIRO Plant Industry. Seeds were germinated on wet paper towel and cultivated at 4℃ for 5 days, then placed at 12℃ for 5 day for germination. Germinated seedlings were grown at 22℃ in a greenhouse. Two-week-old wheat seedlings were treated by different stresses (heat, drought, and heat & drought). The abiotic stress treatments were performed by submerging *T. aestivum* seedling roots in solutions of 37℃ incubator, 25% PEG6000, respectively. *T. aestivum* seedlings treated with water were used as a mock control. The whole *T. aestivum* seedlings were sampled at different time points (0, 1, 6 h). Greenhouse-grown *T. aestivum* plants were used for measuring tissues specific expression patterns of 25 selected *TaC*_*2*_*H*_*2*_*-ZF* genes. Seedling roots (SR), roots at the three leaves (RTLS), roots at the meiosis (RMS), stems at the 1 cm spike (S1S), stems at the two nodes stems (STNS), stems at the anthesis stems (SATS), seedling leaves (SL), leaves at the three tillers (LTTS), leaves at the 2 days after anthesis (L2DAAs), spikes at the two nodes stems (SPTNS), spikes at the meiosis (SPMS), spikes at the anthesis stems (SPAS), grains at the 2 days after anthesis (G2DAAs), grains at the 14 days after anthesis (G14DAAs), grains at the 30 days after anthesis (G30DAAs) were collected. All collected tissue samples were immediately frozen in liquid nitrogen and stored at -80℃ for future analysis.

### Data sets

The annotated genome sequences of *A. tauschii*(*Aegilops tauschii*), *B. distachyon* (*Brachypodium distachyon*), rice (*Oryza sativa Japonica*) and *Arabidopsis(Arabidopsis thaliana)* were downloaded from plantTFDBv5.0, (http://planttfdb.cbi.pku.edu.cn/phylo_tree.php?sp=Tae&fam=C2H2) [60], iTAK software v1.6 (http://itak.feilab.net/cgi-bin/itak/index.cgi) [61], Ensembl plants (http://plants.ensembl.org/Triticum_aestivum/Info/Index). TAIR (Release 10, ftp://ftp.arabidopsis.org/home/tair/Genes/TAIR10_genome_release) and JCVI, (Release 6.1, ftp://ftp.plantbiology.msu.edu/pub/data/Eukaryotic_Projects/o_sativa/annotation_dbs/pseudomolecules/version_6.1), respectively. The partially annotated genome sequences of *T. aestivum* and *Oryza sativa* were downloaded from Prosite (http://au.expasy.org/prosite/) and NCBI (https://www.ncbi.nlm.nih.gov/).

### Identification and characterization of *T. aestivum* C_2_H_2_-ZFPs

For identification of *T. aestivum* C_2_H_2_-ZFPs family members, the information of *T. aestivum* C_2_H_2_-ZFPs sequences was downloaded from the plantTFDBv5.0, iTAK database, and the Ensembl plants. The C_2_H_2_-ZF domain was also queried for Hidden Markov Model (HMM) search through the HMMER 3.0 program. The TaC_2_H_2_-ZF domain was further determined using the SMART database (http://smart.embl-heidelberg.de/) [62].

The theoretical isoelectric point (pI), molecular weight (MW), atomic composition, instability index, aliphatic index, and grand average of hydropathicity (GRAVY) were downloaded from the ExPASy server (http://www.expasy.org/) [63]. The subcellular localization of each TaC_2_H_2_-ZF protein was predicted using the Cell-PLoc 2.0 (http://www.csbio.sjtu.edu.cn/bioinf/Cell-PLoc-2/) webserver [64].

### Classification and chromosomal location of *T. aestivum* C_2_H_2_-ZFPs

The identified 457 TaC_2_H_2_-ZFPs were further manually analyzed to search the numbers of C_2_H_2_-ZF domain, C_2_H_2_-ZF domain sequences, and the space length between C_2_H_2_-ZFPs using the SMART database (http://smart.embl-heidelberg.de/). We defined the C_2_H_2_ type for each identified *T. aestivum* C_2_H_2_ domain, according to the plant-specific amino acid residues and distances between two to nine C_2_H_2_-ZF domains, as have been previously adopted in soybean [3]. Tandem ZF was determined as ZF containing two to thirteen C_2_H_2_-ZF domains, and every two nearby domains are connected by < 12 amino acid residues, such as Tandem ZFs with two subsets assigned as Ta-t1-SF and Ta-t2-SF, respectively. ZF contains one ZF and/or two to four ZF domains each more than 11 amino acid residues between two nearby domains is considered to be scattered ZF. Also, different types of C_2_H_2_-ZFPs have been classified based on the variation of the plant-specific conserved domain “QALGGH” sequence and distances between metal ligands [1, 4, 54]. Generally, C_2_H_2_-ZFP were defined as four types: Q-type, M-type, Z-type, and D-type. C_2_H_2_-ZFP Q-type domains were defined as X2-C-X2-C-X7-QALGGH-X3-H. Those that have 1–5 amino acid degradations in the "QALGGH" domain and that there are certain modifications in the spacing between two cysteine and two histidines of Q type are defined as M-type. Those with more than 12 (Z1) and less than 12 (Z2) in their spacing between the second cysteine and the first histidine were defined as Z-type domains. However, compared with Q-type, M-type, and Z-type, the D-type does not include the second histidine in the C_2_H_2_-ZF domain. According to these defined C_2_H_2_-ZFP types, C_2_H_2_-ZFPs containing a single ZF domain were further classified into four clearly distinguishable subsets (Ta-1i-Q-SF, Ta-1i-M-SF, Ta-1i-Z-SF, and Ta-1i-D-SF). C_2_H_2_-ZFPs containing two Q-type or two M-type C_2_H_2_-ZF domains were defined as Ta-2i-Q-SF and Ta-2i-M-SF, respectively, while the C_2_H_2_-ZFPs containing two different types of C_2_H_2_-ZF domains were classified as Ta-2i-Mix-SF. All ZFPs containing three or four dispersed ZFs were classified into subsets of Ta-3i-SF or Ta-4i-SF, respectively [2, 3].

The *TaC*_*2*_*H*_*2*_*-ZF* genes approximate chromosomal location was determined by blastn search of cDNA sequences against chromosome sequences of *T. aestivum* available at Ensembl Plants (http://plants.ensembl.org/Triticum_aestivum/Tools/Blast). The IWGSC bread wheat genome was assembled using the following parameters: E value ≤ 1e-10 and identity 85% [65].

The chromosomal location image of the *TaC*_*2*_*H*_*2*_*-ZF* genes was generated by TBtools software, according to the chromosomal position information provided in the EnsemblPlants database.

### Analyses of phylogeny, gene structure and conserved motif

For the phylogenetic investigation, multiple alignments were performed with the domain sequences of the TaZFP family proteins by the neighbor-joining (NJ) method available via MEGA7 software [66] to construct an unrooted phylogenetic tree. Sequences were aligned using the MUSCLE alignment algorithm [66]. The tree obtained was the consensus of 1000 single trees provided by bootstraps, gaps/missing data treatment: partial deletion, model/method: LG model, rates among sites: gamma distributed with invariant sites (G + I).

For the *TaC*_*2*_*H*_*2*_*-ZF* gene exon–intron structure, we used the Gene Structure Display Server (GSDS 2.0) (http://gsds.cbi.pku.edu.cn) [67]. We analyzed the additional motifs outside the C_2_H_2_ domain of 204 C_2_H_2_-ZFP amino acid sequences using the Multiple Em for Motif Elicitation (MEME) suite Version 5.1.1 online program (http://meme-suite.org/tools/meme). The optimized parameters of MEME were employed as the following: maximum number of motifs, 10; minimum motif width, 6; and maximum motif width, 50.

### Chromosomal distribution and synteny analysis of *TaC*_*2*_*H*_*2*_*-ZF* genes

The *TaC*_*2*_*H*_*2*_*-ZF* genes were mapped to the chromosome according to the chromosomal location given from the Ensemblplants database by using CIRCA (OMGenomics, http://omgenomics.com/circa/). Investigating the gene duplication events of having the Default parameters using the Multiple Collinearity Scan toolkit (MCScanX) [68].

To exhibit the syntenic relationship of the orthologous *C*_*2*_*H*_*2*_*-ZF* genes obtained from *T. aestivum* and *A. tauschii*, *B. distachyon*, *Arabidopsis*, *O. Japonica* the syntenic analysis maps were constructed using the Dual Systeny Plotter software (https://github.com/CJ-Chen/TBtools) [68]. Synonymous (Ks) and non-synonymous (Ka) substitution rates were calculated for each duplicated *C*_*2*_*H*_*2*_*-ZF* genes using a maximum likelihood model averaging in Ka/Ks Calculator [68]. For each gene pair, the divergence time of collinear gene pairs was calculated using the mean Ks values from T = Ks/(2λ × 10^–6^) Mya (λ = 6.5 × 10^–9^) [69].

### Expression analysis of *C*_*2*_*H*_*2*_*-ZF* genes in *T. aestivum*

The expression profiles of 198 *TaC*_*2*_*H*_*2*_*-ZF* genes in different tissues (roots, stem, leaves, spikes, and grains) and different development stages were determined based on the RNA seq data available from Wheat Expression Browser powered by expVIP (http://www.wheat-expression.com/) [70]. Briefly, the raw data for RNA-Seq were downloaded, and subsequent data processing was performed to remove weakly expressed genes using the R software package. Finally, the gene expression heatmap was generated by using the TBtools view software.

### RNA extraction and qRT-PCR analyses

Total RNA from different tissues (root, stem, leave, spike, grain) and various stress treated materials were extracted using Trizol reagent (Invitrogen) following the manufacturer’s instructions. Subsequently, RNA integrity and quality were checked by electrophoresis of RNA on 1.2% denaturing agarose gel. For qPCR analysis, first-strand cDNA synthesis was performed with the SuperScript II Reverse Transcriptase (Invitrogen) by the manufacturer's instructions. 25 tissue-specific *TaC*_*2*_*H*_*2*_*-ZF* genes expression patterns and 18 *TaC*_*2*_*H*_*2*_*-ZF* genes significantly in response to heat and drought stresses expression patterns were determined by qRT-PCR analysis. Gene-specific primer pairs in Additional file 1: Table S4. Accuracy and specificity of primers were checked by the Blast algorithm and their products were electrophoretically checked on 1.5% agarose gel for amplification accuracy. *TaRP15* (Additional file 1: Table S4) was used as the internal reference gene when examining gene expression of 25 selected *TaC*_*2*_*H*_*2*_*-ZF* genes. the qRT-PCR analysis was performed to detect relative mRNA expression levels. During the qRT-PCR analysis, each qRT-PCR analysis was repeated three times, and data analyzed by analysis software based on the comparative 2^−ΔΔCT^ method of relative gene quantification [3]. Relative expression of 25 selected *TaC*_*2*_*H*_*2*_*-ZF* genes and probability value (P) were also calculated using the qRT-PCR analysis software.

## Supplementary Information

**Additional file 1: Table S1.** Classification of the 457 *Triticum aestivum *C2H2-ZFPs according to the organization of their contained C2H2 fingers. **Table S2.** List of 204 *Triticum aestivum *C2H2-ZFP genes and their related information. **Table S3.** One-to-one orthologous relationships between *T.aestivum *and *T.aestivum*, *T.aestivum *and *A.tauschii*, *T.aestivum *and *B.distachyon*, *T.aestivum *and Rice, *T.aestivum *and *Arabidopsis*. **Table S4.** Primers used for qRT-PCR. **Table S5.** Expression profiles of *T. aestivum *C2H2-ZFP genes.

**Additional file 2:** The full size and high resolution of Figure 3.

**Additional file 3:** The full size and high resolution of Figure 6.

## Data Availability

The datasets analysed during the current study are available in the bread wheat Genome Project (http://plants.ensembl.org/Triticum_aestivum/Tools/Blast), plantTFDBv5.0 (http://planttfdb.cbi.pku.edu.cn/phylo_tree.phpsp=Tae&fam=C2H2), iTAK software v1.6 (http://itak.feilab.net/cgi-bin/itak/index.cgi), Ensembl plants (http://plants.ensembl.org/Triticum_aestivum/Info/Index). TAIR (Release 10, ftp://ftp.arabidopsis.org/home/tair/Genes/TAIR10_genome_release) and JCVI (Release 6.1, ftp://ftp.plantbiology.msu.edu/pub/data/Eukaryotic_Projects/o_sativa/annotation_dbs/pseudomolecules/version_6.1) repository.

## Abbreviations

C_2_H_2_-ZFPs: C_2_H_2_-type zinc finger proteins
*T. Aestivum*: *Triticum aestivum*
*A. tauschii*: *Aegilops tauschii*
*B. distachyon*: *Brachypodium distachyon*
rice: *Oryza sativa Japonica*
*Arabidopsis*: *Arabidopsis thaliana*
CDS: Coding sequence
pI: Isoelectric point
MW: Molecular weight
GRAVY: Grand average of hydropathicity
NJ: Neighbor-joining
GSDS: Gene Structure Display Server
MEME: Multiple Em for Motif Elicitation
MCScanX: Multiple Collinearity Scan toolkit
SR: Seedling roots
RTLS: Roots at the three leaves
RMS: Roots at the meiosisstems
S1S: At the 1 cm spike stems
STNS: At the two nodes stems
SATS: Stems at the anthesis stems
SL: Seedling leafs
LTTS: Leafs at the three tillers
L2DAAs: Leafs at the 2 days after anthesis
SPTNS: Spikes at the two nodes stems
SPMS: Spikes at the meiosis
SPAS: Spikes at the anthesis stems
G2DAAs: Grains at the 2 days after anthesis
G14DAAs: Grains at the 14 days after anthesis
G30DAAs: Grains at the 30 days after anthesis
qRT-PCR: Quantitative real-time PCR
TFs: Transcription factors
